# Exploring community engaged research experiences and preferences: a multi-level qualitative investigation

**DOI:** 10.1186/s40900-021-00261-6

**Published:** 2021-03-30

**Authors:** Hae-Ra Han, Ashley Xu, Kyra J. W. Mendez, Safiyyah Okoye, Joycelyn Cudjoe, Mona Bahouth, Melanie Reese, Lee Bone, Cheryl Dennison-Himmelfarb

**Affiliations:** 1 The Johns Hopkins University School of Nursing, Baltimore, MD USA; 2 The Johns Hopkins Institute for Clinical and Translational Research, Community Engagement Program, Baltimore, MD USA; 3 The Johns Hopkins University Bloomberg School of Public Health, Baltimore, MD USA; 4 Government Accountability Office, Washington, DC USA; 5 The Johns Hopkins University School of Medicine, Baltimore, MD USA; 6 Older Women Embracing Life, Baltimore, MD USA

**Keywords:** Community engaged research, Qualitative, Group interviews

## Abstract

**Background:**

Community engagement may make research more relevant, translatable, and sustainable, hence improving the possibility of reducing health disparities. The purpose of this study was to explore strategies for community engagement adopted by research teams and identify areas for enhancing engagement in future community engaged research.

**Methods:**

The Community Engagement Program of the Johns Hopkins Institute for Clinical and Translational Research hosted a forum to engage researchers and community partners in group discussion to reflect on their diverse past and current experiences in planning, implementing, and evaluating community engagement in health research*.* A total of 50 researchers, research staff, and community partners participated in five concurrent semi-structured group interviews and a whole group wrap-up session. Group interviews were audiotaped, transcribed verbatim, and analyzed using content analysis.

**Results:**

Four themes with eight subthemes were identified. Main themes included: Community engagement is an ongoing and iterative process; Community partner roles must be well-defined and clearly communicated; Mutual trust and transparency are central to community engagement; and Measuring community outcomes is an evolving area. Relevant subthemes were: engaging community partners in various stages of research; mission-driven vs. “checking the box”; breadth and depth of engagement; roles of community partner; recruitment and selection of community partners; building trust; clear communication for transparency; and conflict in community engaged research.

**Conclusion:**

The findings highlight the benefits and challenges of community engaged research. Enhanced capacity building for community engagement, including training and communication tools for both community and researcher partners, are needed.

## Plain ENGLISH summary

Involving communities in the research process can make better the way research is planned, carried out, and used. With growing interest and support for community engagement, it is important to understand the views and insights of people who experienced community engaged research. To explore the key lessons learned by community engaged research teams, we held five group interview sessions with 50 research investigators, research staff, and community partners. Our findings showed that community engagement is not static but a dynamic, ongoing process. Community partners felt that involving them earlier and in all aspects of the research process would make for better science. Researchers were often torn between “checking the box” to meet community engagement requirements set by the funder of their research and engaging community partners in various stages of research to advance the scientific mission because of time pressure. There were strong themes around clearly defined community partner roles as well as mutual trust and transparency, as they were considered central to successful engagement of communities in research. Related, participants noted that conflict between the researchers and community partners is a familiar part of the community engaged research process. Two common sources of tension were misaligned research priorities between researchers and community partners and lack of communication about study results. Lastly, there was little agreement between researchers about how to measure community engaged research impact outcomes or which impact outcomes matter the most. Our findings support the need for training and communication tools for both community and researcher partners.

## Introduction

Community engagement is defined as the process of meaningfully involving communities affected by a research finding in the research process [1]. Community engagement in research is recognized as a key process to improve the way the research is prioritized, translated, and used in a real-life setting, and can reduce health disparities [2–5]. Community engagement can occur across all stages of research including identifying study topics, planning and designing the study, strengthening recruitment strategies, collecting and analyzing data, and interpreting and disseminating findings. Several United States federal health agencies including the National Institutes of Health and Patient-Centered Outcomes Research Institute offer funding for community and other stakeholder engaged research, highlighting growing interest and support at the national level [6].

While evidence regarding the methods of community engagement is increasing, detailed information about the role and scope of community engagement or specific approaches to successful community engagement across the full spectrum of the research cycle is still limited [7]. Additionally, a systematic review of clinical trials that report patient engagement for the purposes of research revealed that an estimated less than 1% of clinical trials engage patients in the research process and that engagement of minorities occurred in only about a quarter of trials [8]. Growing interest in the participation and contributions of community involvement make it an opportune time to examine the key success strategies adopted by research teams and other lessons learned, and to consider the implications for future community engaged health research.

One of the goals of the National Institutes of Health Clinical and Translational Science Awards (CTSA) program is to promote knowledge translation by engaging patients and communities in the research process. The Community Engagement Program of the Johns Hopkins Institute for Clinical and Translational Research—Hopkins CTSA—hosted a forum to engage researchers and community partners in a dialogue to reflect on their past and current experiences in a variety of aspects of community engaged research*.* This paper reports the main themes identified from semi-structured group discussions among diverse forum participants in relation to their past and current experiences in planning, implementing, and evaluating community engaged research*.*

## Methods

### Participants and setting

Group discussions were chosen to identify norms of research teams in their conduct of community engaged research. The forum was publicized to researchers, research staff, and community partners within the greater Maryland-Washington region through email invites using existing lists and word of mouth. In order to ensure diversity in our forum participants, we also sent out personal, verbal, and email invitations to 100 researchers and community partners conducting community engaged research. The forum was also publicized during the public announcement section of meetings hosted by community advisory boards and local agencies. A total of 36 researchers and 14 community members participated in five concurrent group discussion sessions. Researcher participants consisted of research staff (e.g., research program coordinators, research assistants), post-doctoral fellows, and faculty investigators. Community participants included patient consultants and prior study subjects. About 86% of forum participants indicated that they were involved in a community or other stakeholder engaged research project at the time of the forum, and 59% had prior exposure to community engaged research.

### Procedures

A planning committee was formed to develop the goals, agenda and format of the forum. The planning committee included key faculty and staff from the Hopkins CTSA. Also included were members of the Johns Hopkins Community Research Advisory Council—a research review committee consisting of community residents, representatives of local community organizations, and community advocates. The planning committee met over a 3-month time period for a total of 12 meetings and developed forum goals and objectives as well as format, content, and discussion methods. The planning committee set the main goal of the forum to bring together investigators, patients, community members, and other stakeholders to share their experiences working together on research that addresses health and social issues that impact Greater Baltimore, Maryland. The 3-h forum began with opening by Director of Johns Hopkins Institute for Clinical and Translational Research, followed by the keynote presentation by a director of one of the health disparities research centers at the Johns Hopkins University. Participants were then asked to join one of five breakout groups to discuss the following topics: 1) identification and selection of community partners; 2) community partner roles and responsibilities; 3) approaches to promoting community engagement; 4) process and impact evaluation of engagement; and 5) scope of community engagement (see Table 1 for example questions). The forum planning committee grouped the participants into five breakout discussion groups based on their topical preferences, past experiences, and their expertise that were collected during registration. Following the breakout sessions, forum participants reconvened and representatives from each breakout group briefly summarized their discussion and presented the key themes of their respective breakout group discussion.

**Table 1:** Main topics and example questions addressed by each breakout group

Group	Main topic	Example questions
1	Identification and selection of community partners	How do you identify and engage community partners in the research process?
2	Community partner roles and responsibilities	How do you define the role of community partners in health research?
3	Approaches to promoting community engagement	What tangible skills does a researcher need to engage community partners in the research process?
4	Process and impact evaluation of engagement	How do you acknowledge and measure the impact of community engagement?
5	Scope of community engagement	Community engaged research: What is working and what is not working—Community partners speak out

Four of the five breakout groups included both researchers and community members. The fifth group included community members only in order to maximize comfort and sharing of relevant experiences by community members. Each breakout group included 7–11 members and had a moderator to facilitate the discussion (except for the community member-only group which had two co-moderators—one community leader and one researcher), a note taker to transcribe key discussion points for the facilitation of post breakout discussion report out, and two recording devices to record discussion content. Moderators were all well-established researchers with prior and/or current community engaged projects. They had experiences in working with community members and had prior experiences in moderating group discussions. The moderators had specific instructions with a semi-structured discussion guide to follow in order to maximize the exchange of information and facilitate productive discussion. The note takers were all doctoral students who had previous experiences in qualitative research. They were all briefed and trained on the forum purpose and methods. The duration of each group discussion was 1 h. Forum participants provided written permission to audio record the discussion and transcribe notes. The Johns Hopkins Institutional Review Board considered this a quality improvement project and waived it from a full review.

### Analysis

Each group discussion was audio recorded and transcribed verbatim by the original note takers. Following transcription, qualitative content analysis was performed by identifying common themes across group discussions. A standard theme-based content analysis approach was used to analyze the discussion results [9]. Relevant phrases and statements from each group discussion were identified. Phrases and concepts expressed by more than one participant were considered validated and were included in the analysis, with all of the validated phrases and concepts sorted into thematic groups according to similarity. The transcriptions were then read multiple times, key phrases that provided specific information relevant to the research questions were highlighted, and key themes were identified and supported by direct quotes.

## Results

We identified four themes and eight subthemes from the forum. Main themes included: Community engagement is an ongoing and iterative process; Community partner roles must be well-defined and clearly communicated; Mutual trust and transparency are central to community engagement; and Measuring community engagement outcomes is an evolving area. Each theme with accompanying subthemes are detailed in the following section.

### Community engagement is an ongoing and iterative process

Forum participants indicated that the amount of time community partners were engaged and the process of engaging community partners were different at various stages of research. The perceived importance and desire to be involved in the designing and planning stage was discussed more frequently than the desire to be involved in the other stages. Engaging in early phases of research was important to identify a problem and formulate the appropriate research questions. We identified three subthemes in relation to community engagement process: Engaging community in various stages of research, mission-driven vs. checking the box, and breadth and depth of engagement.

#### Engaging community partners in various stages of research

“We should be engaged in all stages of the research process” was a universal and oft-repeated sentiment within the Community partner group. Community partners felt researchers should be cognizant that community engagement is an iterative process, and that researchers’ ability to include community members and other stakeholders in all aspects of the research process is the key to success. In particular, community partners expressed that involving them earlier in the research process would make for better science:

*“I think the framework is critical. The way that the research question is framed is critical. And I think that the community and the specific community, should be involved in developing the research question. Not too many folks would find fault with efforts to improve that disease or whatever may be, but if it’s not framed so that it applies …*” (Participant 6; Member of community research advisory council)

Researchers discussed actively engaging community partners in research through a series of ongoing, interactive process. Having open community forums (i.e., local forums of residents and community groups to identify issues faced by particular communities and neighborhoods and work together to address those issues) was one method to get community partners on same page, understand the needs of the community, and develop trust and rapport with the community. One researcher noted:

*“I will say that the iterative process can actually be big advantage … I think that’s an incentive at least for most of the individuals that I have worked with that they really appreciate how their ideas have taken shape and how their input has been utilized. I think that can make things take a little bit more time but ultimately it is beneficial.”* (Participant 1; Research investigator)

#### Mission-driven versus checking the box

Engaging community partners in all stages of research, however, was challenging at times. Participants discussed the importance of community engagement to advance the scientific mission. Yet, researchers stated that some grant mechanism requirements seem to have a list of community engagement requirements throughout the research study that may not always be productive to the project nor respectful of community partner time. Researchers noted that they do not want to waste community partners’ time unless there is a clear need for their feedback building on their skillset or life experiences. Valuing participant time was highlighted as one of the most difficult but important aspects of conducting research with community partners:

*“I think the most challenging part of our current research is for the patients that I pushed so hard to get, for them to care about this really high level, you know, methodologic question … We don’t have monthly meetings … We try to call on them for mission driven things … We are very strategic about what we ask for [community partners] to provide input on … we don’t just waste their time just for the sake of checking a box.”* (Participant 3; Research investigator).

#### Breadth and depth of engagement

Overall, community engagement was centered around identifying a research question or problem. Participants agreed that having communities identify research questions or problems is the most effective and pragmatic way of conducting community-based research. This process would ensure community buy-in when researchers decide to plan future studies in these same communities. One community member remarked on the good back and forth communication between community members and researchers present when she participated in a group of people living with high blood pressure that consulted researchers on relevant research questions:

*“So that meant a lot to us for the fact, OK, you’re listening, and you’re actually developing something that’s going to, you know, cause I think what they did was they did something that was a consensus of what all of us had said. So we were really encouraged by it, and so when it comes time to actually do the study we want to be a part of the study, you know.*” (Participant 4; Patient).

### Community partner roles must be well-defined and clearly communicated

Researchers expressed the need to consider the role of the community partners before beginning the research process—what is a community partner, the role of community partners, and the best ways to identify and recruit them. There were two subthemes directly addressing these questions: Roles of community partners and recruitment and selection of community partners.

#### Roles of community partners

Often, a bidirectional relationship with the community helped researchers determine the role of community partners. Participants noted that it is important to distinguish the role of community partners, as they are the liaisons that bring the researchers into the community while also acting as advisors, decision makers, and validators. Some participants called a community partner, the *“mayor of the block,”* the person that people in the community go to or someone who would be recognized by the community, and could “hold their own” in discussions about the community. This person would assist in translating what is going on in the community and monitor checks and balances.

The researchers in this discussion underscored the importance of clear communication about each community partner’s role to assure use of common language and clarity of roles in order to optimize the partnership and research. A research staff member talked about the importance of clarity in communication about the community partner roles by stating:

*“We can be clear … I feel sometimes that there is a sense of, um people because they [community partners] don’t know what’s expected of them, feeling like they are not doing what they are supposed to be doing or that they’re we’re not...so we want to avoid that.”* (Participant 9; Research staff).

#### Recruitment and selection of community partners

Community participants discussed the various ways they first became involved in research and collaborated with research teams. A common theme was engagement in research as a form of advocacy for a medical condition of interest. A community partner, the parent of a child with autism, shared her experience:

*“I knew about* *clinicaltrials.gov**, discovered a trial, participated in that trial and then subsequently asked to share my PHI [protected health information] for further research purposes and that was sort of the first time that I felt like I was asked by the research community to share information about my son’s autism and how it affects our family and so forth.”* (Participant 11; Parent of a patient).

From the researcher perspective, it was important to first identify the type of community partner that the study requires and then to discuss who is the individual community member. Funding announcements, dissemination and implementation strategies, and knowing the skill sets of the individual community members were useful for selecting community partners. Nevertheless, difficulty identifying the right people to serve as community partners was a common challenge identified by researchers. Participants acknowledged the importance of relying on community resources and various stakeholders to identify and recruit community partners. For example, working with spiritual leaders and health departments, as well as getting to know and building trust with a community helped to identify community partners. To this end, participants noted that it would be ideal to the research team to establish presence and courtship to the community of interest and establish a relationship. Being active, involved, and partnering with community-based organizations would increase exposure and, in turn, enhance community partner engagement.

“*Having a conversation early on about what are your networks and really documenting that and understanding the kinds of networks that everyone brings to the table and how you can connect with those kinds of individuals or groups so that you can have those relationships built in advance so that when you get to the end of the process you can talk about your findings, you are not scrambling. You’ve established that.”* (Participant 13; Research staff).

### Mutual trust and transparency are central to community engagement

Participants noted that central to conducting community engaged research is the need to develop trust and value the unique contributions of the community partners who are invested in the project. The need to develop trust between researchers and community partners was a universal priority for forum participants. Subthemes to discuss trust to promote community engagement were: building trust, clear communication for transparency, and conflict in community engaged research.

#### Building trust

Participants stressed the importance of building trust long-term with the community and not coming to the partnership without consideration of community partners’ agendas. Building trust among community members and other stakeholders was also noted as an important aspect of conducting ethical and effective health research:

*“That does make a huge difference … when the community sees somebody there, not with their hands out but actually wanting to be there month in and month out so when you do come calling or knocking or you need support, you have the stakeholders that relationship built that you can go to the head, the leadership of the community and they know you and they trust you.”* (Participant 8; Member of community advisory council).

#### Clear communication for transparency

Participants underscored that researchers must make the research process as transparent as possible to community members. This included clear, honest and transparent communication with community members about funding, study findings, study team commitment to the community, duration of the study, and the overall goals of the study. Some community members felt, however, there was a lack of information from researchers to participants regarding results of the study.

*“They very often don’t even let you know what, why they collected it, and how it impacted the analysis and then what they’re going to do with it. We never hear that part …”* (Participant 5; Patient and member of community research advisory council).

#### Conflict in community engaged research

Researchers acknowledged that conflict between the researchers and community partners is a familiar part of the community engaged research process. Two sources of tension discussed by community partners were misaligned research priorities between researchers and community partners and lack of communication about study results. Researchers and community partners noted, however, that conflict was not always reported. When it was reported, it was not always clear how to manage conflict:

*“I’ve been thinking a lot about [conflict] in many different [ways], but … as with muscles and anything, it is essential for growth and you need pain and destruction to move on. That’s how you know how you exercise well. When your muscles are torn and they need to regrow and repair. Otherwise you haven’t worked out enough... It’s the same for group engagement ... So how to manage [conflict] I don’t know but … that’s key.”* (Participant 17; Research investigator).

### Measuring community engagement impact outcomes is an evolving area

Researchers discussed a variety of community engagement outcomes they believed should be measured, such as participant attendance at meetings or activities, community partner needs, conflicts and conflict resolution, the amount of money and funding raised by community partners, and community partner self-efficacy. The researchers acknowledged it is easier to measure and evaluate short-term community engaged research outcomes like impact on study design rather than impact on health or impact of community engaged research on a community. However, they noted the lack of a commonly accepted impact measurement framework to guide the measurement of community engaged research for its impact. There was little agreement between researchers about how to measure community engaged research impact outcomes or which impact outcomes matter the most. Community engaged research might lack a commonly accepted impact measurement framework because it is an emerging field or as a result of differing goals of engagement that guide the evaluation of impact outcomes between projects. In the discussion about impact measurement, a researcher stated:

*“What if we did this on the principle of justice? How would you measure justice? We get back to what you said about the goals. The goal is to incorporate justice, and that’s really why we are doing it. Can you measure something like that or do you want to be democratic. Or how would you measure whether your process was democratic and just or to some extent inclusiveness? It’s very hard to measure these types of things.”* (Participant 20; Research investigator).

## Discussion

Researchers and policy-makers alike increasingly recognize the importance of seeking diverse and inclusive perspectives in translational research. Nonetheless, limited information is available about the role and scope of community engagement or specific approaches to community engagement across the full spectrum of the research cycle [7]. In particular, this paper offers the diverse perspectives of research investigators, staff, and community partners actively involved in community engaged research. This forum discussion allowed these diverse forum participants an opportunity to share their experiences and perspectives about the benefits and challenges of community engaged research. Our participants noted that community engagement is an ongoing and iterative process to which mutual trust and transparency are central and that the roles of community partners must be well-defined and clearly communicated for the engagement to be successful. These main themes are overall consistent with the key principles of engagement (i.e., reciprocal relationships, partnerships, co-learning, and transparency-honesty-trust) as highlighted in the recent literature [7, 10–12].

Whereas all forum participants recognized the benefits of community engaged research, some of the subthemes such as engaging community in various stages of research suggest the need for closer dialogue between researchers and community partners in earlier phases of research. It was interesting to note that researchers felt engagement should happen less, once the research started; it was important for them to not waste community partners’ time by focusing on “mission driven things.” We did not find a similar concern about time burden among community partners. An essential element of community engaged research is the meaningful participation of a broadly representative group of stakeholders whose contributions are sought through all phases of the research, beginning with the planning and research question [12–14]. Indeed, the researcher participants in the forum noted that the most effective community engaged research involved community partners to identify a problem and formulate the appropriate research questions. Some of the data driven approaches such as the discrete choice experiment—a quantitative technique to uncover how individuals value selected attributes of a program by asking them to state their choice over different hypothetical alternatives [15]—may be useful to elicit community preferences as a way of enhancing their engagement in the early phase of research. For example, a recent systematic review [16] revealed that the discrete choice experiment, when applied to designing and characterizing therapies in the planning phase of research, resulted in increased acceptability and appropriateness.

Engagement in “all stages of the research process” came through as a strong theme within the Community partner group. Community partners in the forum appreciated the “back and forth” interaction between researchers and community members. A mixed methods study [17] showed that researchers do not routinely give feedback to community partners. Yet, community partners who receive feedback are motivated for further engagement as they feel it supports their learning and development while prompting researchers to reflect on the impact of community partners [17]. One of the ways in which researchers can provide such feedback would be to bring study findings back to the community—a lacking area in the research process, as noted by the community members. A survey of 109 community partners involved in health research with academic institutions [18] reported “research results disseminated to the community” as one of the top indicators of successful community engagement. Taken together, these findings suggest the need for culturally relevant and appropriate strategies to promote mutual feedback and better integration of community partners in the research process.

Many of the challenges discussed by the forum participants in the subthemes of mission-driven vs. checking the box, breadth and depth of engagement, and conflict in community engaged research occurred because priorities, motivations, and ways of working differed between researchers and community partners, which caused conflict and power struggles. Some of the practical issues associated with these subthemes (e.g., difficulty recruiting a set of experienced partners well connected to the target community or patient group, long-term commitment needed from partners, and time and cost limits imposed on studies) were recognized in a focus group study [19] in which problems connecting with the right person at the right time, individual member reluctance, and lack of skills and training were identified as main barriers to stakeholder engagement.

As suggested by our participants, clear and transparent communication is central to resolving potential conflicts in community engaged research. In particular, transparency in communication between community partners and researchers in terms of budget and research administrative processes [20] has been noted as key factors for improved community engaged research partnerships. Additionally, shared training opportunities for community partners and researchers may be helpful to build community partner skills about research and facilitate engagement on both sides [19]. The subtheme of recruitment and selection of community partners underscores the importance and need for the development of community capacity to facilitate more meaningful engagement in research. At the policy level, community engaged research needs to build in appropriate time, and funders should acknowledge this need as part of providing an appropriate context and budget for community engagement, to create the conditions where engagement has the potential to have a positive impact.

Forum participants considered a variety of impact assessments for their research and overall reported positive impact of community engagement on research, ensuring its appropriateness and relevance. They noted that it was difficult to have a common impact measure because of differing goals of engagement between projects. Indeed, a recent review of 68 studies addressing measures of community engagement reported that most studies used narrative descriptions of impact data [21]. Similarly, a mixed-methods study in which documents of 200 primary care research projects were examined and 191 researchers were surveyed noted qualitative reporting of community engaged research impact for study processes (e.g., designing methods or developing participant information) or on individual principal investigators (e.g., developing the grant application, managing the research, conducting the research, or the reputation of the principal investigator’s institution) [22]. A qualitative investigation [23] in the United Kingdom also revealed diverse views among stakeholders on what to measure and how to measure their impact. Taken together, the evidence base as to what constitutes adequate impact measures of community engaged research seems evolving. The findings suggest further substantive methodological development in terms of the way in which the impact of community engaged research is measured and reported, a clearer conceptualization of the nature of “impact,” and qualitative and quantitative methods for assessment of impact [23].

There are a number of study limitations to discuss. First, this was a convenience sample that was created for the purpose of the forum. In particular, we targeted researchers and community members who were already supportive of the idea of community involvement in research. Therefore, generalizability of the findings is limited. Additionally, we did not collect detailed sociodemographic information about the forum participants. The interpretation of the qualitative data might have looked different had we had this information such as age, working status (working/retired), or other relevant characteristics (roles, past experiences, etc.). Finally, it is possible that some of the moderators and notetakers of the concurrent discussion groups may not have been independent of the participants in his/her group and might have influenced the discussion either positively or negatively. We attempted to minimize the potential bias and impact of moderators and notetakers on the nature and direction of the discussion in each group by training them prior to the forum and offering them with an interview guide.

## Conclusion

Successful implementation of healthcare interventions relies on community engagement at every stage, ranging from assessing and improving the acceptability of innovations to the sustainability of implemented interventions. In order to optimize the implementation of healthcare interventions, researchers, administrators, and policymakers must weigh the benefits and costs of complex multidimensional arrays of healthcare policies, strategies, and treatments [24]. This cannot be accomplished without meaningful engagement of key community partners throughout the research process. Challenges identified by the study teams underscore the need for enhanced community engagement training, joint planning of engagement activities, agreeing upon community partner roles and expectations in the early-planning stages of the proposed study, and increased opportunities for community participation in the research process.

## Data Availability

The data (anonymized transcripts from the group interviews used for the purpose of this analysis) that support the findings of this study are available from the corresponding author upon reasonable request.

## Abbreviations

CTSA: Clinical and Translational Science Awards
